# Prognostic Awareness in Advanced Disease: A Review Update and Concept Analysis

**DOI:** 10.3389/fpsyg.2021.629050

**Published:** 2021-06-24

**Authors:** Franziska Kühne, Myriel Hermann, Martina Preisler, Amy Rohrmoser, Anne Letsch, Ute Goerling

**Affiliations:** ^1^Department of Psychology, Clinical Psychology and Psychotherapy, University of Potsdam, Potsdam, Germany; ^2^Charité Comprehensive Cancer Center, Charité–Universitätsmedizin Berlin, Corporate Member of Freie Universität Berlin, Humboldt-Universität zu Berlin, Berlin Institute of Health, Berlin, Germany; ^3^Internal Medicine II, Hematology and Oncology, University Medical Center Schleswig-Holstein, Kiel, Germany

**Keywords:** prognosis, advanced disease, cancer, oncology, palliative care, patient-centered care, systematic review

## Abstract

**Purpose:**

Although subjective knowledge about the prognosis of an advanced disease is extremely important for coping and treatment planning, the concept of prognostic awareness (PA) remains inconsistently defined. The aims of the scoping review were to synthesize a definition of PA from the most recent literature, describe preconditions, correlates and consequences, and suggest a conceptual model.

**Methods:**

By using scoping review methodology, we searched the *Web of Science* and *PubMed* databases, and included publications, reviews, meta-analyses or guidelines on all physical diagnoses, as well as publications offering a conceptual or an operational definition of PA. The data were analyzed by means of content analysis techniques.

**Results:**

Of the 24 included publications, 21 referred exclusively to cancer, one to patients with hip fractures and two to palliative care in general. The deduced definition of PA comprised the following facets: adequate estimation of chances for recovery, knowledge of limited time to live, adequate estimation of life expectancy, knowledge of therapy goals, and knowledge of the course of the disease. Further content analysis results were mapped graphically and in a detailed table.

**Conclusion:**

There appears to be a lack of theoretical embedding of PA that in turn influences the methods used for empirical investigation. Drawing on a clear conceptual definition, longitudinal or experimental studies would be desirable.

## Introduction

The concept of PA has received increased attention within health care research in recent years. In 2000, the term was used by Chochinov et al. (2000) as the acknowledgment of an advanced medical diagnosis in order to prepare for an imminent death. Jackson et al. (2013) understand PA “as a patient’s capacity to understand his or her prognosis and the likely illness trajectory” (p. 894). From a theoretical perspective, in their common sense model of self-regulation of health and illness, Leventhal et al. (1998) outline the role of so-called representations (i.e., individual definitions of illness) for coping behaviors. They assume various different dimensions of such representations: the disease *label* (e.g., cancer) and its symptoms (e.g., breast lump), the *timeline* (regarding the development of the disease, its duration and recovery), perceptions of *causes*, and *consequences*, as well as *control* beliefs (e.g., disease assessed as preventable, curable or possible to prevent its progress). The common sense model also highlights the importance of emotions, either as part of illness representations themselves, or as a response to them (Diefenbach and Leventhal, 1996). Referring to this model, PA could be regarded as a specific component of the timeline dimension. Nonetheless, PA is mostly viewed as an independent construct, with a rather weak theoretical embedding.

PA is assumed to be associated with better quality care, i.e., earlier hospice and palliative care, and fewer resuscitations (Jackson et al., 2013). As PA may support patients in adapting medical care and personal decisions to their needs, values and goals, it is highly relevant (Jackson et al., 2013). One strategy to promote PA is through patient-practitioner-communication, specifically the empathic exploration of patients’ knowledge, and the subsequent transfer of information in a way that helps patients to manage and integrate the given prognostic information (Jackson et al., 2013).

Recently, Applebaum et al. (2014) defined PA as the “awareness of a terminal prognosis or shortened life expectancy” of palliative patients (p. 1103). From a systematic review, they deduced the following facets of PA: (a) awareness of a metastatic, advanced or terminal disease, (b) awareness of shortened life expectancy or the specific likelihood of survival and (c) awareness of the purpose of treatment (Applebaum et al., 2014). The primary studies included in the review involved between one and all of these aspects, which hampers comparisons and resulted in the large range (0–75%) of patients described to show adequate PA (Applebaum et al., 2014). Despite its influence on the research field, the review focused on cancer and on the measurement of PA, and the authors presented their results only narratively. The search was completed in 2012, which is why the review does not cover recent studies, whereas publications on PA increased especially during the last 5 years.

Therefore, the aims of our current update were to (i) derive a definition of PA from the most recent literature, (ii) describe preconditions, correlates and consequences, and based on the results, (iii) suggest a conceptual model of PA in advanced disease in general.

## Materials and Methods

The review was conducted in accordance with the Preferred Reporting Items for Systematic Reviews and Meta-Analyses guidelines (Moher et al., 2009; PRISMA flow diagram available upon request from the corresponding author). As we aimed to update and complement the narrative review of Applebaum et al. (2014), we decided on a scoping review methodology. Such a review is a systematic, but economic evidence synthesis, focusing on central concepts and an overview of the current state of research (Levac et al., 2010; Armstrong et al., 2011; Colquhoun et al., 2014).

### Search and Inclusion Criteria

The electronic search was conducted in two key psychosocial and medical databases, i.e., *Web of Science* and *PubMed*. To refer to the current evidence, we built on the previous search (Applebaum et al., 2014) by starting our search on 01 January 2013, and defining the search date itself (15 February 2019) as its end point. We further decided to extend the previous search terms, and thus combined the term prognose^∗^ with each of the following concepts: aware^∗^, know^∗^, attitude^∗^, and understand^∗^. We included peer-reviewed original publications, reviews, meta-analyses and guidelines written in English. In order to take a broad perspective, we did not restrict the search to a specific physical diagnosis, to cancer or to adults. Publications with a conceptual definition, description or explanation of PA, but also those using an operational definition of PA were eligible for inclusion.

### Screening and Selection

A reviewer (MH) screened the search results for titles and abstracts, so as to exclude records that were clearly irrelevant (e.g., biological or technical papers). Then, the reviewer retrieved the full-texts of the remaining records and screened them for inclusion. Disagreements were resolved by discussion with a second team member during regular meetings (FK or UG). Since we expected a broad spectrum of definitions, we refrained from connecting definitions with quality ratings, and thus decided against the assessment of study quality.

### Concept Analysis

For the concept analysis, we referred to the approach proposed by Scholl et al. (2014), and used MAXQDA (2020) software. Referring to content analysis techniques (Mayring, 2014; Kuckartz, 2018), MH inductively derived sub-categories for the main categories *definition*, *preconditions*, *correlates*, and *consequences*. Preconditions and consequences were extracted from longitudinal studies or experimental and intervention designs only. If one aspect was relevant to two or more categories, it was assigned to all relevant categories. In order to concentrate on relevant topics, we established a new sub-category (see subheadings under 3.1.-3.4.) if an issue was mentioned in at least two publications. Since only a limited number of publications mentioned preconditions and consequences, i.e., concerned causal relations, we changed our procedure, and included any entry that referred to a precondition or a consequence. To enhance the analysis, a coding guideline with a description of each category and illustrative examples was developed. Again, disagreements were resolved by discussion with a second team member (FK or UG).

In a second step, another team member (LPW) familiarized herself with the data, and then independently classified all units of meaning into the category system by using the coding guide. Inter-rater agreement was *κ* = 0.85; values > 0.70 are assumed to be appropriate (Wirtz, 2017). Finally, a definition was proposed and the categories were mapped conceptually, including the preconditions, correlates and consequences of PA (see Supplementary Material 1).

## Results

*N* = 24 publications were included; two of them were systematic reviews, and one was a theoretical paper (Supplementary Material 2). 16 of the original publications used cross-sectional designs, four were longitudinal or intervention studies, and one study used qualitative methods. Whereas, 21 publications dealt with cancer patients, one referred to patients with hip fractures and two included a palliative population with various diagnoses. In 20 publications, the stage of the disease was described as terminal, advanced or metastasized.

### Definition of PA

According to our content analysis, *PA primarily comprises the appropriate estimation of chances for recovery (i.e., incurable disease), knowledge of limited time to live and the appropriate estimation of shortened life expectancy, and secondarily, the appropriate estimation of therapy goals as well as knowledge of the course of a disease.* Below, each component of PA is outlined further. Relations between concepts are mapped in Supplementary Material 2.

#### Appropriate Estimation of Chances for Recovery

With *n* = 16 (66.7%) of entries (Diamond et al., 2014, 2017; El-Jawahri et al., 2014, 2015; Tang et al., 2014, 2016a,b, 2018; Shin et al., 2016; Chen et al.,2017a,b, 2019; Kurita et al., 2018; Mack et al., 2018; Sato et al., 2018; Janssens et al., 2019), this was one of the most important components of PA. Often (*n* = 11), patients with advanced disease were only considered to have accurate PA if they viewed their disease as incurable (Diamond et al., 2014, 2017; Tang et al., 2014, 2016a,b, 2018; Chen et al.,2017a,b, 2019; Sato et al., 2018; Janssens et al., 2019); other authors (*n* = 4) asked patients to rate their chances of recovery in percent (0–100%; El-Jawahri et al., 2014, 2015; Shin et al., 2016; Mack et al., 2018). All patients who stated 0–10% chances of recovery and/or whose view was concordant with their physician’s assessment were then classified as having adequate PA. Furthermore, in two publications, knowing the exact stage of the disease was also rated as having adequate PA (Chen et al., 2017a; Kurita et al., 2018).

#### Knowledge of Limited Time to Live

This category (*n* = 15, 62.5%) refers to patients’ knowledge of the proximity of death, i.e., knowledge about a life-limiting disease, death approaching in near future, or considering a disease as terminal (Diamond et al., 2014, 2017; El-Jawahri et al., 2014; Tang et al., 2014, 2016a,b, 2018; Enzinger et al., 2015; Fisher et al., 2015; Chen et al.,2017a,b, 2019; Nipp et al., 2017; Kurita et al., 2018; Shen et al., 2018).

#### Appropriate Estimation of Life Expectancy

Ten publications (41.7%) considered this aspect as important to PA (Diamond et al., 2014, 2017; Liu et al., 2014; Enzinger et al., 2015; Fisher et al., 2015; McLawhorn et al., 2016; Chen et al., 2017a; Eikelboom et al., 2018; Kurita et al., 2018; Shen et al., 2018). For reconciliation, physician’s assessments or statistical values were used.

#### Appropriate Knowledge of Therapy Goals

Six publications (25%) included this component of PA (Jackson et al., 2013; El-Jawahri et al., 2014; Fisher et al., 2015; Chen et al., 2017a; Nipp et al., 2017; Sato et al., 2018), and the subjective goal of therapy was often dichotomized (i.e., curative vs. non-curative; El-Jawahri et al., 2014; Fisher et al., 2015; Chen et al., 2017a; Nipp et al., 2017; Sato et al., 2018), and the latter was associated with adequate PA.

#### Knowledge of Course of Disease

In three publications (12.5%), this aspect of PA referred to both a more general view and to specifics such as the expected physical level of functioning (Jackson et al., 2013; McLawhorn et al., 2016; Eikelboom et al., 2018).

### Precondition: Readiness/Preference for and Obtained Information

Only one publication (4.2%) was considered relevant for this category (Chen et al., 2019). It explained that the patients’ readiness for prognostic information and the information they actually received (via conversations with physicians or family) were associated with more adequate PA.

### Correlates of PA

#### Time Between Diagnosis and Death

Eight publications (33.3%) dealt with the role of time, i.e., more time passed since diagnosis, and proximity to death were correlated with higher PA (Liu et al., 2014; Tang et al., 2014, 2016a,b; Enzinger et al., 2015; Chen et al., 2017b, 2019; Janssens et al., 2019). To the contrary, another publication considered a more advantageous prognosis with an expected positive course as associated with higher PA (Mack et al., 2018).

#### Mental Health

Higher PA was associated in four studies (16.7%) with more depressiveness (El-Jawahri et al., 2015; Shin et al., 2016; Nipp et al., 2017; Sato et al., 2018), and in three studies (12.5%), with increased anxiety (El-Jawahri et al., 2014; Nipp et al., 2017; Sato et al., 2018).

#### Quality of Life

In all eight publications (33.3%) that contributed entries to this category, more adequate PA was associated with lower emotional, physical and social quality of life (El-Jawahri et al., 2014, 2015; Fisher et al., 2015; Tang et al., 2016a; Nipp et al., 2017; Kurita et al., 2018; Sato et al., 2018; Janssens et al., 2019). One of those publications indicated that high PA was associated with higher existential quality of life (Fisher et al., 2015).

#### Quality of Treatment

Four studies (16.7%) described a correlation between high PA and more care conversations (e.g., advanced care planning) as well as receiving less aggressive treatments (Tang et al., 2014, 2016b; Enzinger et al., 2015; Shen et al., 2018).

#### Readiness/Preference for and Obtained Information

Four studies (16.7%) characterized a more pronounced readiness/preference of patients for information and open communication of prognostic information by health care practitioners and caregivers as associated with higher PA (Diamond et al., 2014; Liu et al., 2014; Tang et al., 2014; Enzinger et al., 2015).

#### Context Characteristics

The authors of three publications (12.5%) described differences in PA dependent on country (Chen et al., 2017a), region (Tang et al., 2014), or site (Tang et al., 2014; Fisher et al., 2015). The frequency of adequate PA was rated highest in Australia and lowest in southern Europe and the United Kingdom (Chen et al., 2017a).

#### Patient Characteristics

According to three studies (12.5%), accurate PA was correlated with younger age (Tang et al., 2014) higher educational level (Tang et al., 2014), higher cognitive capacity (Fisher et al., 2015), and also with unemployment (Sato et al., 2018). One study each described female (Sato et al., 2018) or male (Tang et al., 2014) gender as correlated with higher PA. In one study, lung cancer patients were described as more likely to adequately understand their prognosis (Tang et al., 2014).

### Consequences of PA

#### Quality of Treatment

In two publications (8.3%), the more adequate the PA, the more conversations about care were conducted and the less aggressive treatments patients received (Tang et al., 2018; Chen et al., 2019).

#### Depressiveness

The authors of one publication described depressive symptoms as a consequence of more accurate PA (El-Jawahri et al., 2015).

## Discussion

The purpose of this scoping review was to update and refine the definition of PA, derive its preconditions, correlates and consequences from the current literature, and then to suggest an empirically based conceptual model. Given our results, and in line with Applebaum et al. (2014), PA represents a multi-faceted construct. Awareness of a metastatic, advanced or terminal disease (facet a) turns up in the present categories of the appropriate estimation of chances for recovery and the knowledge of limited time to live. The shortened life expectancy or the likelihood of survival (facet b) is reflected in the adequate estimation of life expectancy. Knowledge of the purpose of treatment (facet c) resembles the current appropriate estimation of therapy goals. Furthermore, understanding the future course of the illness is viewed as a component of PA. Thus, our scoping review confirms and refines previous results. Thus, by drawing on recent primary studies, the review supports the consistency of the definition.

Contrary to the previous review (Applebaum et al., 2014), our study highlights the role of mental health (symptoms of depression and anxiety) and of quality of life, which were both negatively correlated to PA. In line, active coping strategies are important for mental health in advanced disease (Nipp et al., 2017). On the other hand, our results confirm that proximity to death plays a crucial role in the development of PA, and that adapted conversations about prognosis and treatment options are particularly important for high-quality care. Furthermore, the needs and wishes of palliative patients concerning their extent of participation in decision-making may vary considerably, depending for example on the type or stage of the disease, cultural background or emotional distress (Enzinger et al., 2015). If patients and their caregivers prefer a shared involvement in decision-making, both seem to benefit in terms of improved knowledge about care goals, advanced care planning and treatment options (Fisher et al., 2015). The information preferences of patients and caregivers may vary considerably too; with caregivers tending to vaccinate between open conversations and the desire to maintain hope for their patients (Applebaum et al., 2018).

Some of the included studies suggested demographic and regional effects, that is, PA was correlated with younger age, higher educational level and cognitive capacity of patients. Nonetheless, the broader evidence on shared decision-making points to the significance of patient beliefs and expectations about their role and expertise in determining involvement of patients in decision-making (Janssens et al., 2019). These authors conclude from their comprehensive review that the power imbalance between physicians and patients, and also the perceived acceptability of patient involvement are more important than individual variables (e.g., age, culture) *per se*, and even than patients’ information preferences. They call for a change in attitudes so as to foster shared decision-making (Janssens et al., 2019). Above, PA may be considered within the concept of health literacy, which basically refers to “people’s knowledge, motivation and competences to access, understand, appraise, and apply health information […]” (Sørensen et al., 2012, p. 3).

As a limitation to our review, most included studies used correlational designs. The results on the preconditions and consequences stem from four studies only, so that they must be interpreted with caution. The majority of studies stemmed from cancer populations, thus it is too early to generalize our results to other diseases. One of the few longitudinal investigations of PA reveals little change in patient and caregiver PA during the progression of illness, but clearly, more such research is necessary (Liu et al., 2014). Experimental studies manipulating PA in analogue samples may complement our understanding of underlying mechanisms. In addition to qualitative interviews, quantitative studies using brief measures of illness perceptions (Broadbent et al., 2006) or on treatment preferences (Mack et al., 2018) may add to the PA literature.

Although we used a structured and systematic approach, we focused our resources, and concentrated on two common literature databases. Since one reviewer screened for inclusion, regular team meetings were scheduled in order to discuss decisions. Unlike previous reviews on the topic, we referred to structured, qualitative methods to analyze the data, indicated inter-rater agreement, mapped the results in a detailed table, and depicted them graphically. PA seems to comprise knowledge about incurability and shortened life-expectancy, but also an adequate understanding of the course of the disease and the therapy goals. By clarifying the construct, the review contributes to a broader understanding of PA. Obtaining stakeholder views on the definition proposed would be useful, to guide future research.

## Author Contributions

FK, MH, UG, MP, and AL had the idea for the article. MH performed the literature search. FK, MH and UG analyzed the data. All authors drafted the manuscript and critically revised the work.

## Conflict of Interest

The authors declare that the research was conducted in the absence of any commercial or financial relationships that could be construed as a potential conflict of interest.

## Abbreviations

PA: prognostic awareness.
