# Supplementary material for: Prognostic Awareness in Advanced Disease: A Review Update and Concept Analysis
Source: Front Psychol. 2021 Jun 24;12:629050. doi: 10.3389/fpsyg.2021.629050 (PMC8264792; doi:10.3389/fpsyg.2021.629050)
Supplement: Supplementary file 1 [file Data_Sheet_1.PDF]

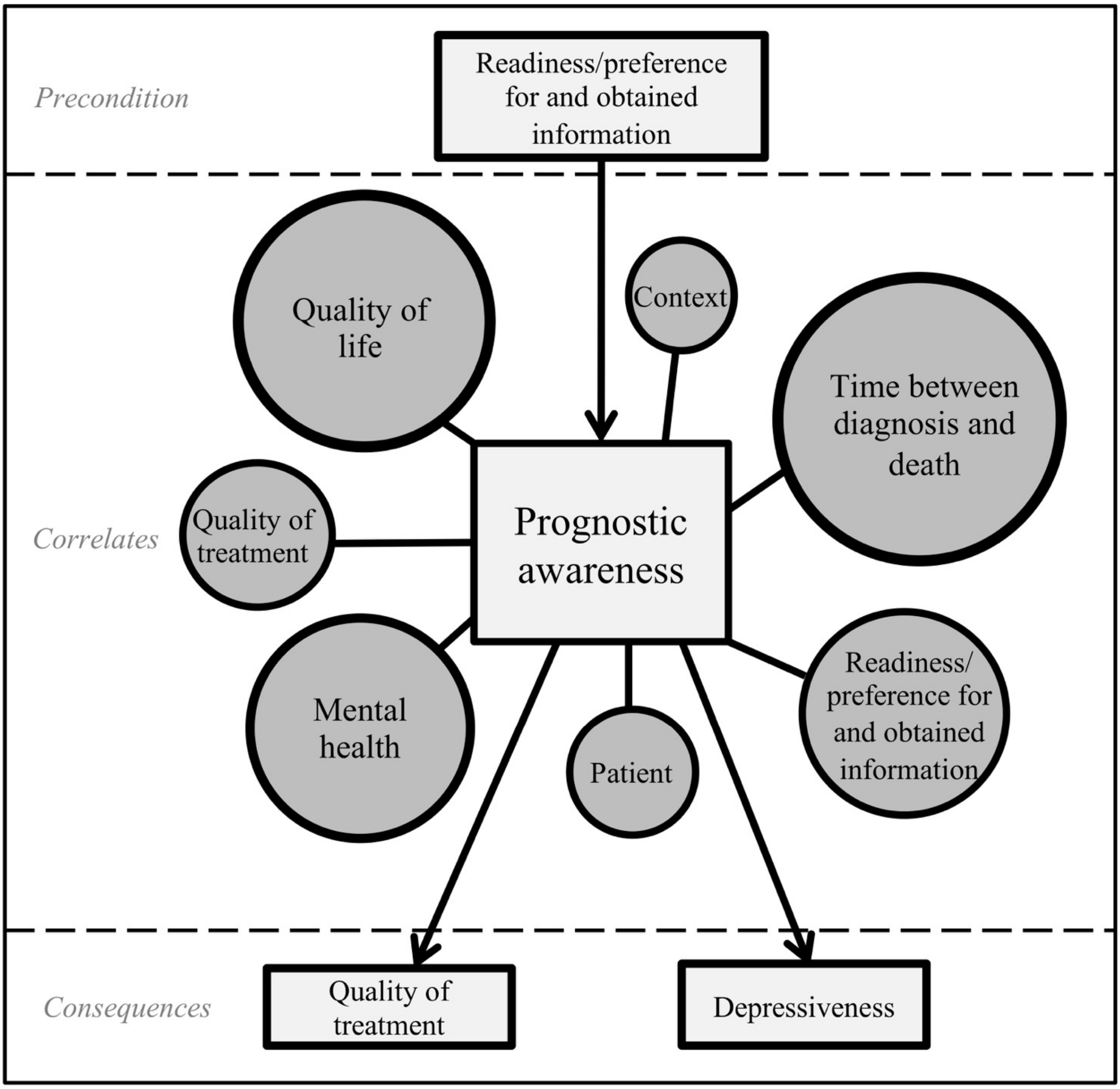

Supplement 1. Conceptual model of prognostic awareness (larger circles and frames indicate stronger impact due to the number of entries in a category).
