# Supplementary material for: Prognostic Awareness in Advanced Disease: A Review Update and Concept Analysis
Source: Front Psychol. 2021 Jun 24;12:629050. doi: 10.3389/fpsyg.2021.629050 (PMC8264792; doi:10.3389/fpsyg.2021.629050)
Supplement: Supplementary file 2 [file Data_Sheet_2.PDF]

Supplement 2. Detailed overview of PA definitions and of related concepts.

**Study**	**Definition**	**Relations**	**Categories**
Chen et al. (2019)	Patients' adequate perception that they cannot be cured and will probably die soon.	(+) Information about prognosis and life-sustaining treatments tailored to the readiness for information of the patients.* (+) Facilitation of End of Life Care discussions among patients, caregivers and physicians.*<br>(–) Reduction of life-sustaining treatments (cardiopulmonary resuscitation).**<br>(+) Time proximity to death.	1, 9, 2
Chen et al. (2017a)	Patients with accurate prognostic awareness realistically know and acknowledge:<br>(1) that the goal of treatment is no longer curative, but palliative<br>(2) the stage of their disease (e.g. end-stage)<br>(3) the incurability<br>(4) the terminal nature and the fact that death is approaching and<br>(5) their limited remaining life span.	Association with culture: differences by region (between countries).	7
Chen et al. (2017b)	Patients' adequate perception that they cannot be cured and will probably die soon.	(+) Time proximity to death.	2
Diamond et al. (2014)	Acknowledgement of the incurability and the terminal nature and of the disease and a shortened life expectancy.	(+) Patients' preference to know their prognosis.<br>(–) Caregivers' effort to conceal prognosis.<br>(–) Poor delivery of prognostic information by physicians.	6
Diamond et al. (2017)	Acknowledgement of the incurability and the terminal nature of the disease and a shortened life expectancy.		
Eikelboom et al. (2018)	Patients' adequate perception of the likely future course of their disease (e.g. likelihood of recovery) and their life expectancy.		
El-Jawahri et al. (2015)	Realistic view on the likelihood of cure.	(–) Patients' quality of life (including symptoms of fatigue).<br>(+) Patients' depressive symptoms.<br>(+) Patients' depressive symptoms.**	4, 3, 10
El-Jawahri et al. (2014)	Includes the patient's knowledge about the current goals of therapy, a realistic perception of the likelihood of cure and the terminal nature of their disease.	(–) Patients' quality of life.<br>(+) Patients' anxiety.	4,3

Enzinger et al. (2015)	Patients’ ability to acknowledge the terminal nature of their disease and to adequately estimate their life expectancy.	(+) Prognostic disclosure by physicians (more precisely: patients’ recall of prognostic disclosure by physicians).<br>(+) Patients’ advance care planning.<br>(+) Patients’ preference for comfort-oriented end of life care (vs. life-prolonging care).	6, 5
Fisher et al. (2015)	Acknowledgement of the palliative intent of therapy (vs. curative), the terminal nature of their disease and a shortened life expectancy.	(+) A shorter prognosis.<br>(+) More informal care hours (patients’ loss of independence).<br>(+) Being at peace with life.<br>(–) Patients’ cognitive impairment.<br>Association with site: differences between facilities	2, 4, 8, 7
Jackson et al. (2013)	Ability to understand the likely illness trajectory and to hold an accurate view of the goals of treatment.		
Janssens et al. (2019)	Realistic perception of their likelihood of cure.	(–) Patients’ emotional well-being (as a facet of quality of life).<br>(+) Patients’ physical pain (as a facet of quality of life).<br>(+) Longer time interval from the diagnosis.	4, 2
Kurita et al. (2018)	Patients’ late-stage illness understanding, the acknowledgement of the terminal nature of their disease and the accurate estimation of their life expectancy.	(–) Patients’ perceived physical well-being.<br>(–) Patients’ perceived cognitive functioning.	4
Liu et al. (2014)	Accurate estimation of their life expectancy.	(+) Physicians’ propensity to discuss prognosis.<br>(+) Time proximity to death.	6, 2
Mack et al. (2018)	Patients’ realistic perception of their likelihood of cure.	(+) Good prognosis: favourable anticipated outcomes.	2
McLawhorn et al. (2016)	Capacity to understand the likely illness trajectory and the limited life expectancy.		
Nipp et al. (2017)	Acknowledgement that the intent of therapy is not curative and that the nature of their disease is terminal.	(–) Patients’ quality of life.<br>(+) Patients’ depressive symptoms.<br>(+) Patients’ anxiety.	4. 3
Sato et al. (2018)	Realistic perception regarding the goals of therapy and their likelihood of cure.	(–) Patients’ quality of life.<br>(+) Patients’ depressive symptoms.<br>(+) Patients’ anxiety.<br>(–) Male gender.<br>(–) Patients’ employment (vs. no employment).	4, 3, 8
Shen et al. (2017)	Acknowledgement that the end of life is near and includes the accurate estimation of their life expectancy.	(+) Higher rates of do-not-resuscitate orders.	5
Shin et al. (2016)	Realistic perception regarding their likelihood of cure.	(+) Patients’ depressive symptoms.	3

Tang et al. (2016a)	Acknowledgement, that the disease is not curable and they will probably die in the near future.	(–) Patients' quality of life.<br>(+) Patients' self-perceived sense of burden to others.<br>(+) Time proximity to death.	4, 2
Tang et al. (2018)	Acknowledgement, that the disease is not curable and they will probably die in the near future.	(+) Physician-patient end of life care discussions.**	9
Tang et al. (2014)	Acknowledgement, that the disease is not curable and they would probably die in the near future.	(+) Patients' preference for prognostic disclosure by their physicians.<br>(+) Longer time interval after the diagnosis.<br>(+) Patients' preference for comfort-oriented end of life care (vs. life-prolonging treatments), for less aggressive life-sustaining treatments and hospice care.<br>(–) Female gender.<br>(–) Patients' age.<br>(+) Patients' level of education.<br>Association with type of diagnosis (different types of cancer)<br>Association with site: differences between facilities<br>Association with culture: differences by region (within one country)	6, 2, 5, 8, 7
Tang et al. (2016b)	Acknowledgement, that the disease is not curable and they will probably die in the near future.	(+) Time proximity to death.<br>(–) Patients' preference for intensive care unit care, intubation, and mechanical ventilation.	2<br>5

Notes. * Prerequisites, ** Consequences, (+) = Positive relation, (–) = Negative relation. 1 = Readiness/preference for obtained information (prerequisite), 2 = Time between diagnosis and death, 3 = Mental health, 4 = Quality of life, 5 = Quality of treatment, 6 = Readiness/preference for and obtained information, 7 = Context characteristics, 8 = Patient characteristics, 9 = Quality of treatment (consequence), 10 = Depressiveness (consequence).
